# 

**Published:** 1879-12

**Authors:** 


Whole Number, CCCCXV.

December, 1879.

Number 6, Vol. XXXIX.

THE

### CHICAGO MEDICAL Journal and Examiner

### EDITORS:

WILLIAM H. BYFORD, A.M., M.D.,
JAS. NEVINS HYDE, A.M., M.D. N. S. DAVIS, M.D., LL.D.
DANIEL R. BROWER, M.D.

Chicago Medical Press Association.

Aug. 2D

1875.

CHICAGO:
*PUBLISHED BY THE CHICAGO MEDICAL PRESS ASSOCIATION.*
188 Clark Street.

☞ **Read the Notice of this Journal among Advertisements.**

